# Combined magnetron sputtering and pulsed laser deposition of TiO_**2**_ and BFCO thin films

**DOI:** 10.1038/s41598-017-02284-0

**Published:** 2017-05-31

**Authors:** D. Benetti, R. Nouar, R. Nechache, H. Pepin, A. Sarkissian, F. Rosei, J. M. MacLeod

**Affiliations:** 1 INRS Centre for Energy, Materials and Telecommunications, 1650 Boul. Lionel Boulet, J3X 1S2 Varennes, QC Canada; 2 Plasmionique Inc., 1650 Boul. Lionel Boulet, J3X 1S2 Varennes, QC Canada; 3 School of Chemistry, Physics, and Mechanical Engineering, Queensland University of Technology (QUT), Brisbane, 4001 QLD Australia; 4 Département de Génie Electrique, Ecole de technologie supérieure, 1100 rue Notre-Dame Ouest, Montréal, QC H3C 1K3 Canada

## Abstract

We report the successful demonstration of a hybrid system that combines pulsed laser deposition (PLD) and magnetron sputtering (MS) to deposit high quality thin films. The PLD and MS simultaneously use the same target, leading to an enhanced deposition rate. The performance of this technique is demonstrated through the deposition of titanium dioxide and bismuth-based perovskite oxide Bi_2_FeCrO_6_ (BFCO) thin films on Si(100) and LaAlO_3_ (LAO) (100). These specific oxides were chosen due to their functionalities, such as multiferroic and photovoltaic properties (BFCO) and photocatalysis (TiO_2_). We compare films deposited by conventional PLD, MS and PLD combined with MS, and show that under all conditions the latter technique offers an increased deposition rate (+50%) and produces films denser (+20%) than those produced by MS or PLD alone, and without the large clusters found in the PLD-deposited films. Under optimized conditions, the hybrid technique produces films that are two times smoother than either technique alone.

## Introduction

The growth of thin films and nanostructures can be pursued using a number of technologies, the most successful of which also allow for tailoring the structure so as to optimize optical, electrical, tribological and other properties^1–4^.

These properties, among others, depend critically on the film density, surface morphology^5, 6^ and crystallographic structure. Amongst various deposition technologies, physical vapor deposition (PVD) techniques for thin film production have found widespread use in many industrial sectors. Magnetron sputtering (MS) is a thin-film PVD technique that is widely used to synthesize highly uniform and smooth films^7^ on large surface areas. It offers several advantages compared to thermal or e-beam deposition techniques, since the higher energy in MS atoms can improve adhesion and help to define the deposited film structure. Control over the deposition process could further be improved if the operating pressure of MS, which is typically in the few to tens of mTorr range, could be reduced. The minimum pressure required in a conventional MS system is defined by the ionization rates in the plasma^7^. Operating at higher pressures has a direct effect on the structure and density of deposited films, since the sputtered species lose part of their energy through interaction with the background gas molecules.

Pulsed laser deposition (PLD) is another important PVD technique for thin film and nanostructure growth^1, 8–10^, which can be applied at any pressure, offering more versatility. The ability to control the background pressure and the substrate-to-target distance enables the possibility of controlling the density of the deposited film^11^. However, despite its popularity and widespread use within the research community, the PLD process has faced some challenges related to scaling up for large-area deposition. Another drawback relates to the large clusters that often form during the ablation process^8, 9^; these clusters can introduce undesired inhomogeneity and roughness into the films. Reduction of the pulsed laser energy density helps to reduce or eliminate macroparticle formation, but also lowers the deposition rate.

Several approaches and techniques have been developed to address these challenges relating to film quality in both MS and PLD^12–14^. Unbalanced magnetron sputtering^15^, the high power impulse MS (HIPIMS) process^16^, and plasma assisted PLD^17^ are recent developments that can improve certain characteristics of the deposited films. Techniques to overcome the limitations of PLD for large surface applications are also being proposed and demonstrated^18, 19^.

One promising approach is to combine magnetron sputtering and pulsed laser deposition (MSPLD) into a hybrid system. In most implementations, MSPLD comprises the use of two targets (one for sputtering and one for PLD) that can be used simultaneously^20–24^. This technique has shown promise for the preparation of functional gradient transition metal carbides and multilayer structures in the TiC and diamond-like carbon (DLC) material systems as well as for other nanocomposites^25–29^. Another hybrid approach involved combining PLD with radio frequency (RF) sputtering at the substrate, where the applied RF power can be used to exercise some control over film properties such as composition and crystallinity^30, 31^.

Here, we implement a different hybrid approach that circumvents some of the limitations of traditional MS and PLD by using a single target for both the MS and PLD. Under this hybrid approach, the pulsed laser can be used to trigger and maintain the magnetron discharge at lower pressures, adding another control mechanism for tuning the deposited film structure.

To demonstrate the advantages of the hybrid technique we have studied the growth of TiO_2_ thin films on Si(100), as well as the growth of complex bismuth-based perovskite Bi_2_FeCrO_6_ (BFCO) on LaAlO_3_ (LAO) (100). TiO_2_ is a widely studied semiconducting material with attractive characteristics such as a high refractive index, wide band gap, and photocatalytic properties. BFCO has been recently widely studied due to its multi-ferroic and photovoltaic properties. We have investigated the effects of substrate temperature, operating pressure, pulsed laser and MS power density on the properties of films, and the process has been optimized for power efficiency. Our results show that the hybrid technique leads to improved quality of the deposited films, as well as increasing film uniformity and deposition rate.

## Results and Discussion

**Figure 1:**
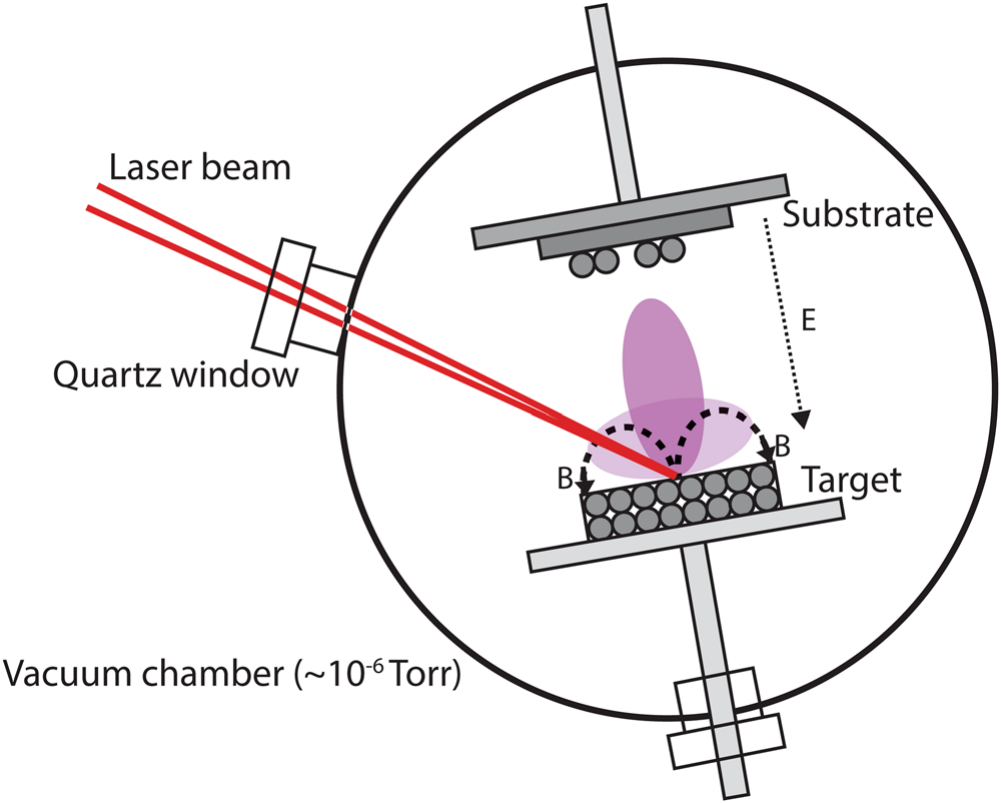
Combined Pulsed Laser Deposition + Magnetron Sputtering System. The target is exposed to the PLD laser (the dark purple vertical plume in the picture) and to the plasma generated by the sputtering system (in light purple in the picture) at the same time. B represents the magnetic field while E is the electric field.

Figure 1 shows a schematic diagram of the hybrid system. In a hybrid PLD/MS system, the plasma plume liberated by the laser pulse triggers and maintains the magnetron discharge at a lower pressure than in a standard MS system. This increased pressure leads to an increase in the plasma density and sputtering rate. Both neutral species and ions pass through the confining magnetic field and are deposited on the substrate, directly influencing the deposited film thickness and structure. The combined PLD/MS system increases the deposition rate through two mechanisms: (1) increased plasma sputtering rate and (2) direct PL deposition of neutral atoms and clusters.

**Figure 2:**
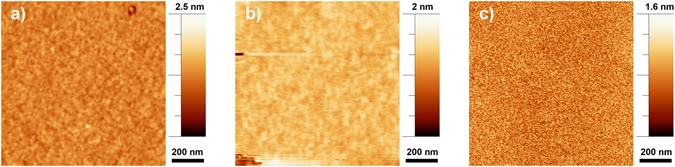
AFM images (on an area of 1 μm × 1 μm.) of the TiO_2_ films realized with the three different techniques (**a**) Magnetron Sputtering (MS), (**b**) Pulsed Laser Deposition (PLD) and (**c**) hybryd technique (MS/PLD) at 5 mTorr.

**Figure 3:**
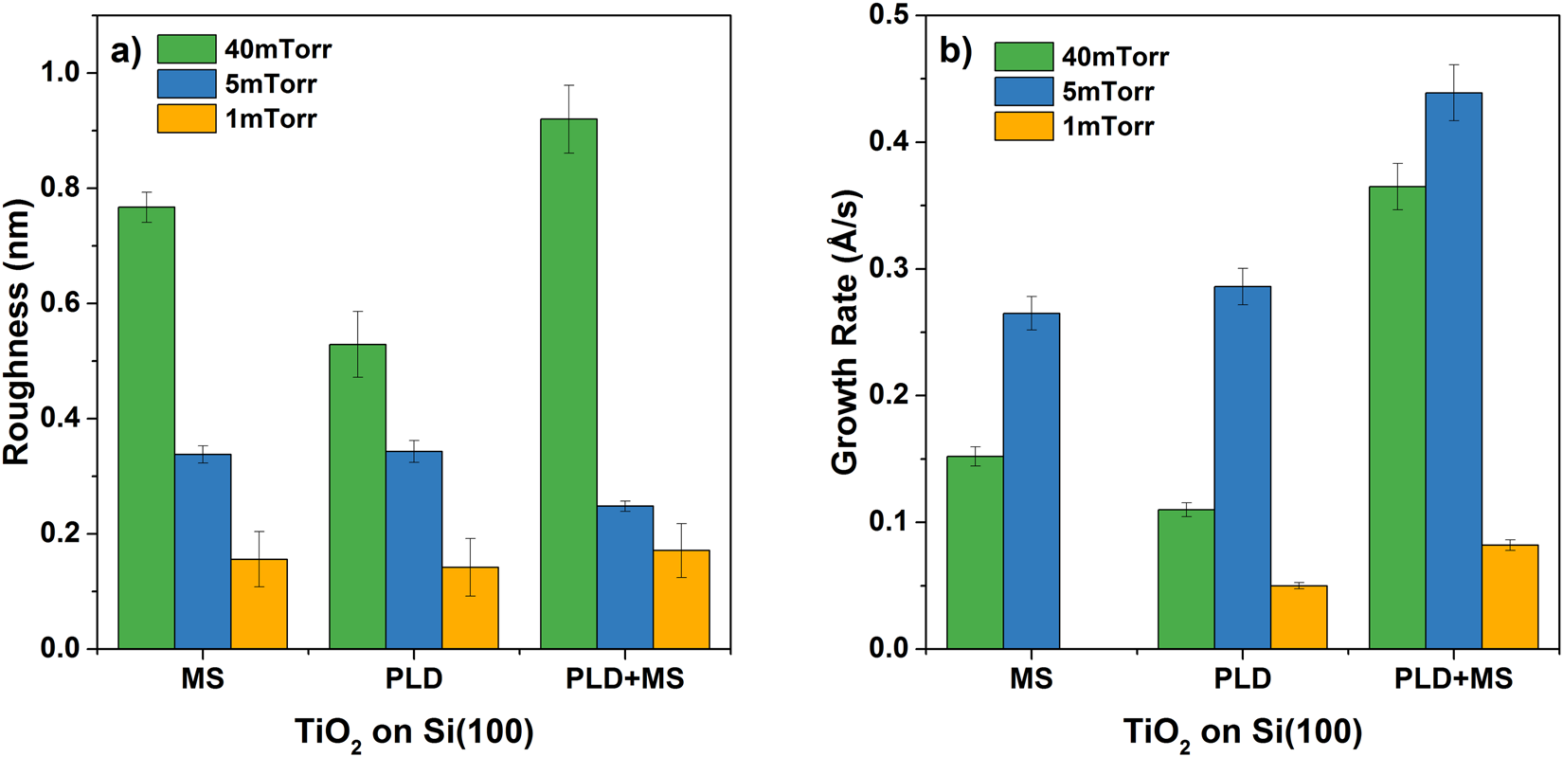
Morphological parameters of thin films. (**a**) AFM-measured roughness of 5 μm × 5 μm TiO_2_ films as a function of argon pressure. (**b**) Growth rate of TiO_2_ films at different values of pressure.

Figure 2 displays representative AFM images of TiO_2_ samples obtained using MS, PLD and the hybrid technique at an operating pressure of 5 mTorr. Regardless of which technique is used, the surface roughness of the TiO_2_ films decreases with decreasing argon pressure (Fig. 3a). Samples fabricated at 1 mTorr, corresponding to the minimum operational pressure, have low roughnesses (~0.18 nm RMS) that are identical within uncertainty for all deposition techniques. However, the growth rates were very slow at 1 mTorr. At low power and pressure, sputtering is inefficient since the collisions are limited, reducing the yield, while the low power limits the movement of the ions. At 5 mTorr the hybrid system consistently operated at a higher growth rate (+50%) while achieving roughness values significantly lower than those obtained through PLD or MS alone (0.25 ± 0.01 nm RMS vs. 0.34 ± 0.02 nm RMS and 0.34 ± 0.02 nm RMS). At higher pressure the surface mobility of the deposited species is reduced due to the impinging argon flux, which manifests in the formation of grains with an average lateral size of 35 ± 4 nm (Figure S2).

**Figure 4:**
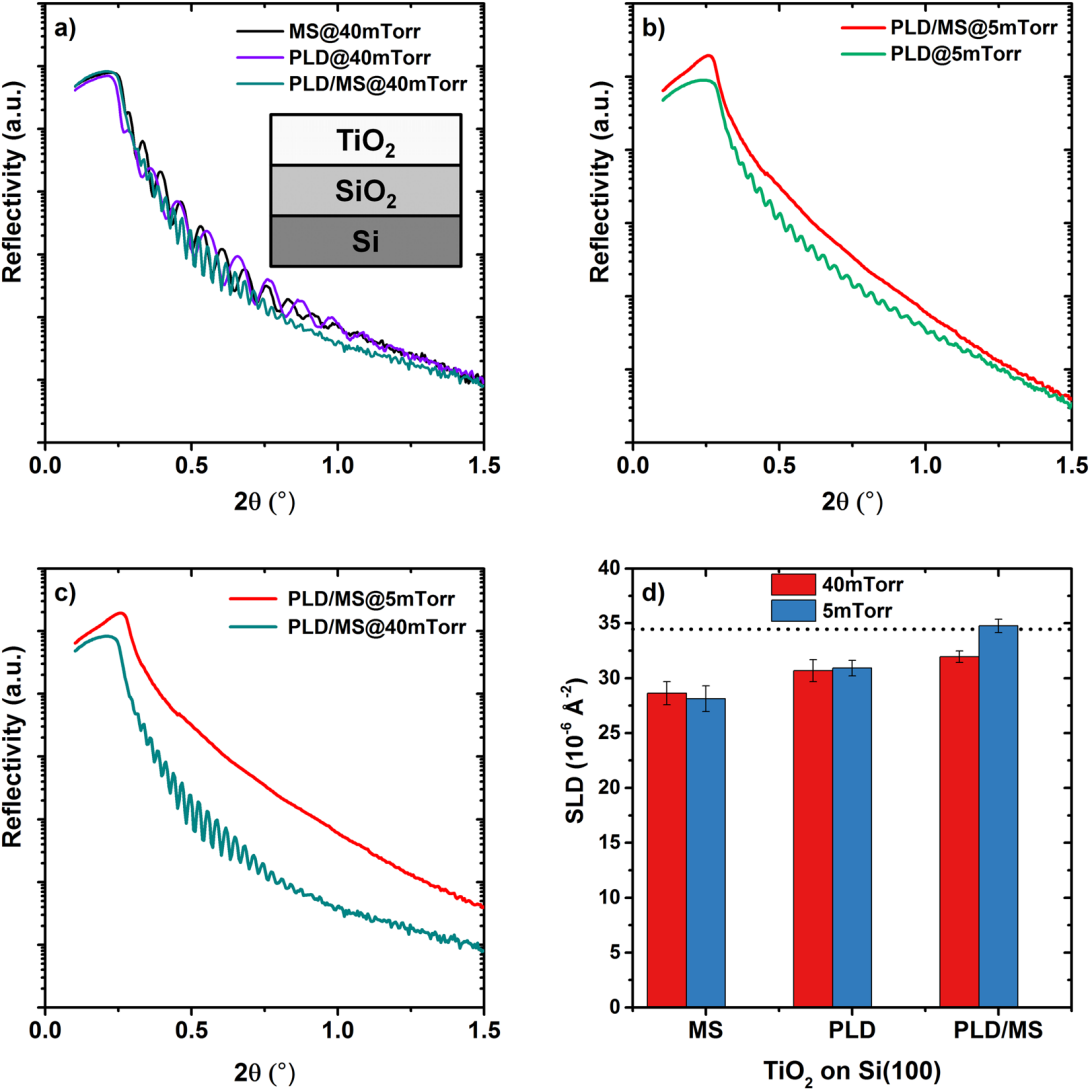
Reflectivity of TiO_2_ samples on Si(100) substrates. (**a**) XRR of the samples deposited at 40mTorr with the three different techniques. In the inset the model used for fitting the data: between the TiO_2_ and Si layers it was necessary to add a thin layer of SiO_2_ (size not in scale), consistent with the presence of a thin native oxide layer at the surface. (**b**) XRR of samples at 5 mTorr. (**c**) Comparison of the samples deposited with hybrid technique at two different pressure, 5 and 40 mTorr. (**d**) Scattering Length Density values for TiO_2_ layers deposited with the three techniques (MS,PLD and hybrid MS/PLD) at two different pressures, 5 and 40 mTorr. The horizontal dashed line represents the tabulated SLD of bulk TiO_2_ (34.46 × 10^−6^ Å^−2^)^33^.

Figure 4 displays the x-ray reflectivity (XRR) data for TiO_2_ samples obtained using different pressures and deposition techniques. The period of oscillation depends on the film thickness, with shorter periods corresponding to thicker films^32^. XRR data also indicate a critical angle, *θ*_c_, which is related to the density of the film. For *θ* *<* *θ*_*c*_ total reflection occurs, whereas above *θ*_*c*_, the XRR signal decreases rapidly with increasing incident angle. The film deposited with the hybrid technique at 5 mTorr exhibits a larger critical angle (Fig. 4b), indicating that it has a higher density compared to the other film.

On average, slower decays in reflectivity were observed for the samples grown with the hybrid technique, indicating that the surface roughness is smaller. An exception is the sample obtained at 40 mTorr with the hybrid technique, which has the largest XRR-determined roughness, consistent with AFM measurements. In general at higher pressure the surface mobility of diffusing species is reduced due to reduced energy of impinging particles, leading to rougher surfaces.

Figure 4d shows the Scattering Length Density (SLD) obtained from analysis of the XRR data for TiO_2_ layers obtained at 40 mTorr and 5 mTorr. The SLD is directly related to the density of the film, with higher values indicating more tightly packed scattering entities. This analysis reveals that independent of the deposition pressure, the hybrid technique produces films with 15–25% higher SLD than MS alone. At the optimal pressure of 5 mTorr the film has the highest value of SLD, which is similar to the tabulated value for bulk TiO_2_ (34.8 vs 34.5 10^−6^Å^−2^)^33^

The hybrid technique was also used to fabricate BFCO films on LAO(100), to investigate whether the use of sputtering combined with PLD could enhance the growth rate and/or uniformity of the film. Since epitaxial growth is necessary to control the crystalline phase and cation ordering of BFCO, which are linked to its functional properties^34^, we focused on obtaining epitaxial film growth using the hybrid technique.

**Figure 5:**
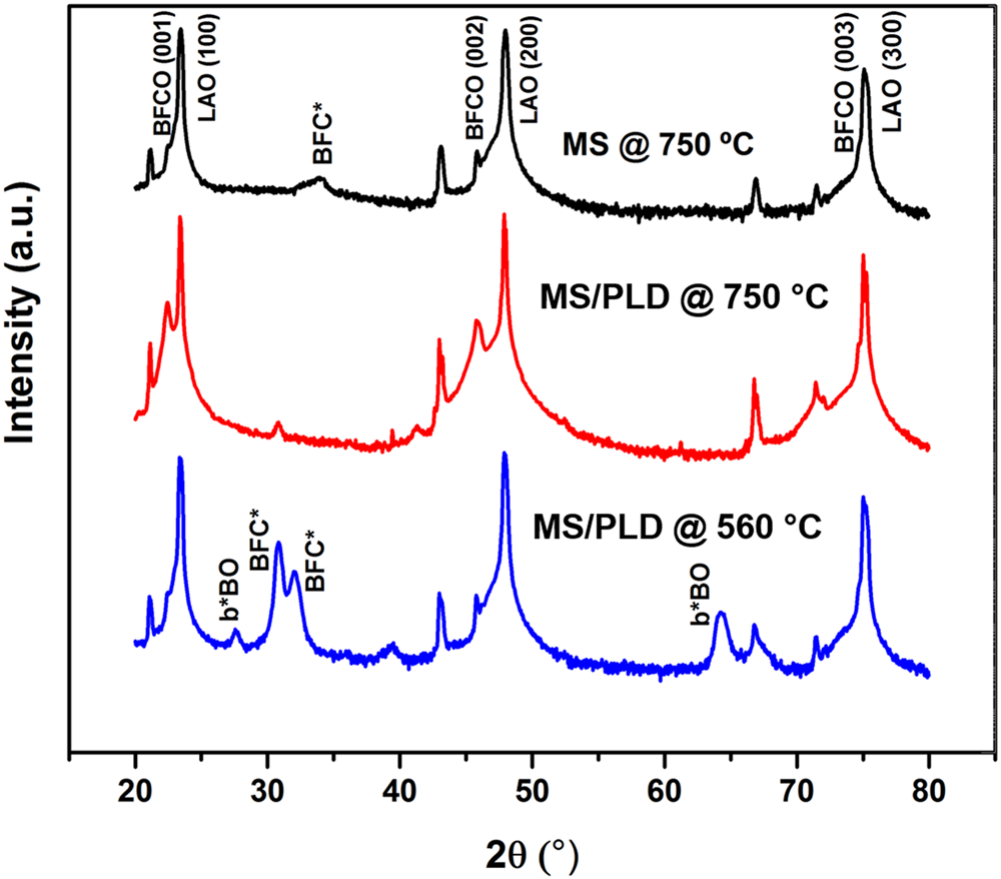
XRD of BFCO samples on LAO (100). Preparation by hybrid MS/PLD at 750 °C is the only route tested that leads to a pure BFCO phase.

Through a systematic variation of parameters, we identified the optimum growth conditions (temperature at 750 °C and pressure at 10 mTorr, laser power 4.4 W and MS discharge 30 W) for which the film presented a smooth surface with an AFM-measured RMS roughness of less than 6 nm. To evaluate the crystallinity of the samples, we measured XRD patterns. Diffraction from samples fabricated on Si(100) did not reveal any crystalline phases. Diffraction from films grown on LAO(100) is shown in Fig. 5.

The sample made with the hybrid technique at 750 °C on LAO(100) produces only diffraction peaks from the substrate and the BFCO layer. Only the 00l (l = 1, 2, 3) cubic reflections of the films are visible, indicating that the films are highly (001)-oriented. These peaks are consistent with data found in literature^35–37^ and confirm the epitaxial growth of the film.

**Figure 6:**
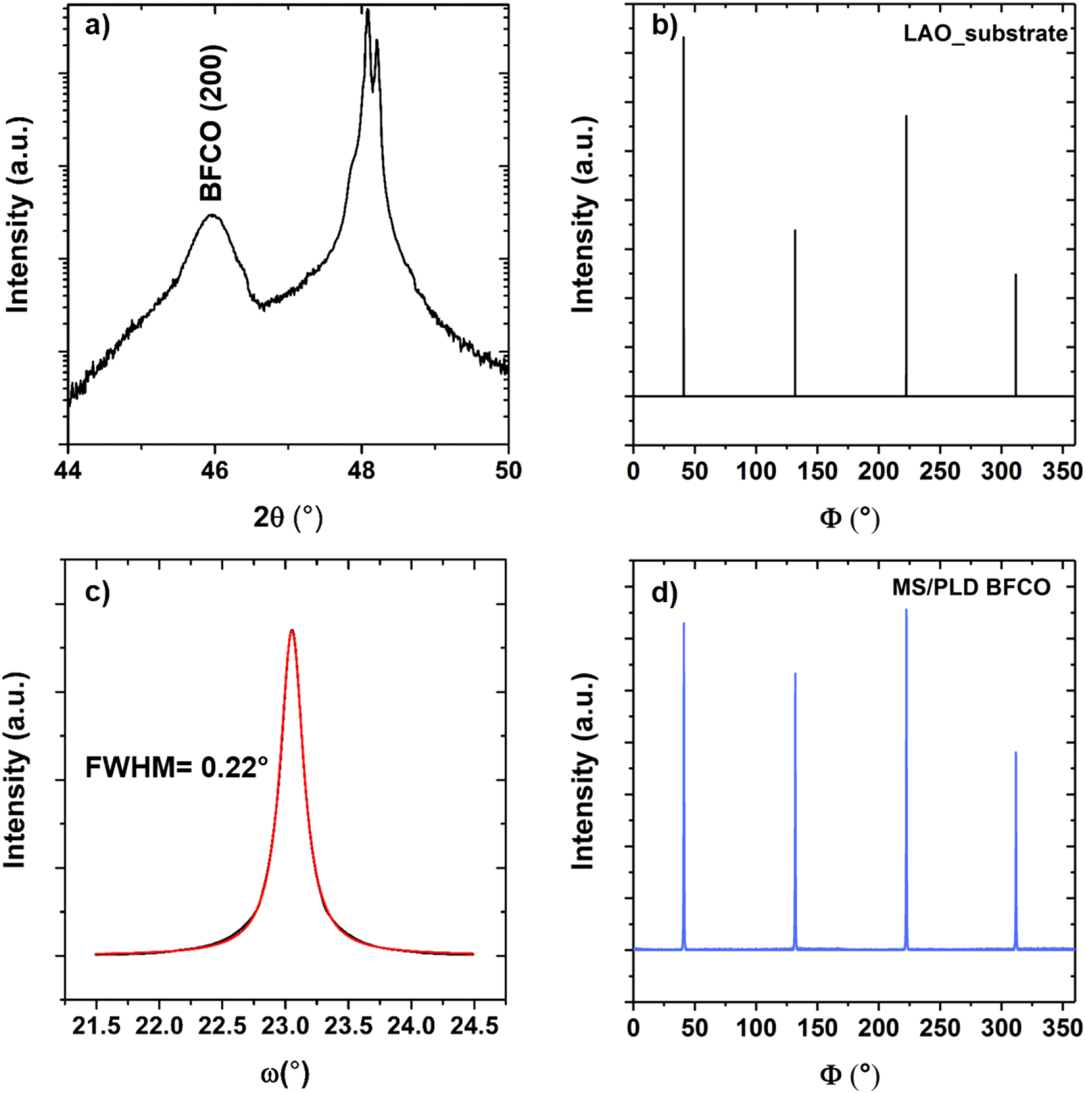
Detailed diffraction data from the BFCO layer grown at 750 °C with the hybrid technique. (a) 2θ-scan and (c) rocking curve of BFCO(002) peak. The red line is a Lorentzian fit to the data (black). Corresponding φ-scan measurements around the (200) reflection on the bare substrate (b) and the BFCO film (d)﻿ confir﻿m the epitaxial growth of the film.

Rocking curve measurements (Fig. 6c) reveal the highly crystalline quality of the BFCO layer, with a full width half maximum (FWHM) of 0.2° around BFCO(002). The epitaxial ordering of BFCO using the hybrid technique is confirmed by the diffraction pattern analysis (φ-scan) reported in Fig. 6b and d, in which we observe a fourfold symmetry of the epitaxial relationship between the BFCO layer and the LAO substrate.

**Figure 7:**
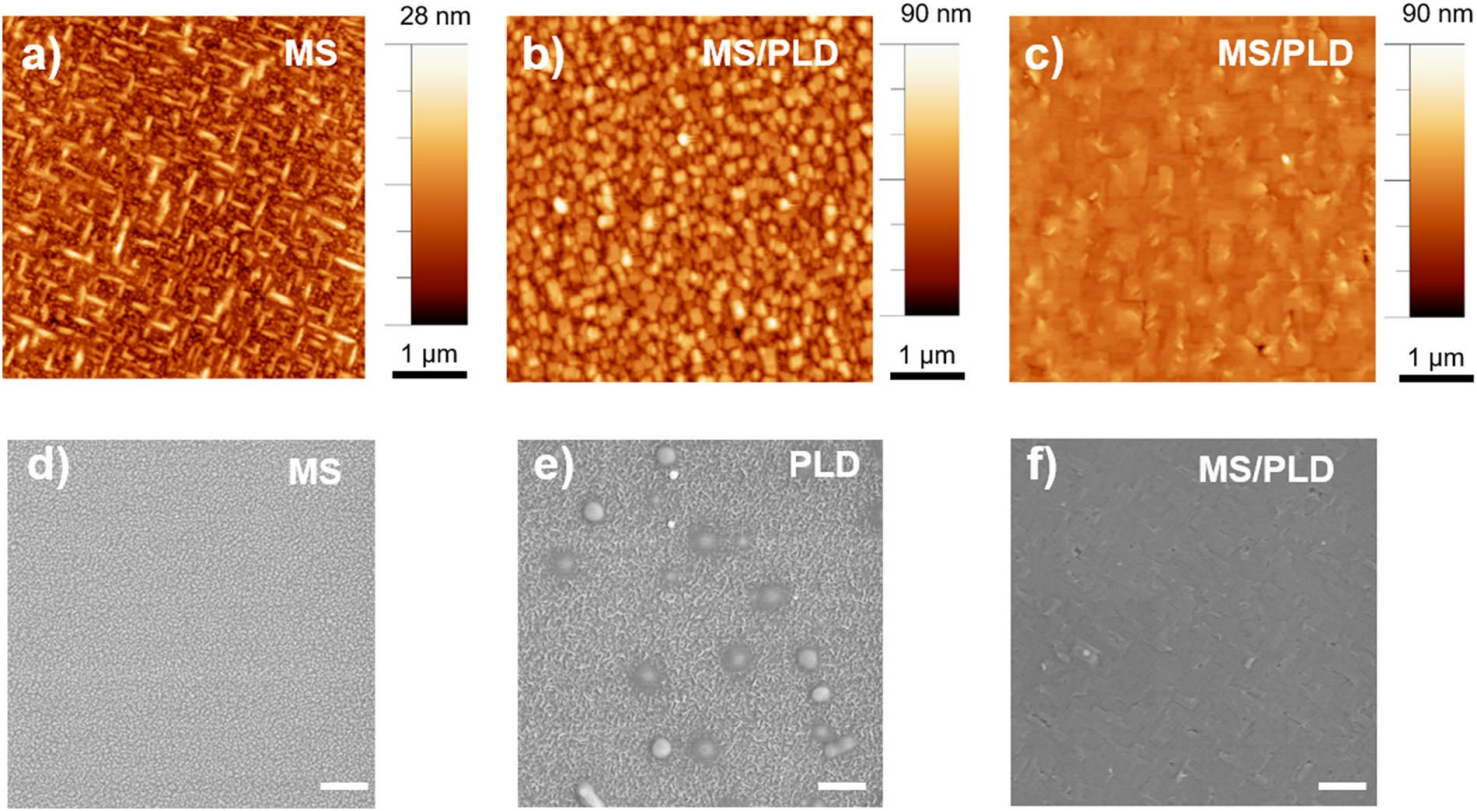
AFM images on an area of 5 μm × 5 μm of different BFCO thin film samples: (**a**) BFCO on LAO(100) deposited with MS at 750 °C, (**b**) BFCO on LAO(100) deposited with MS/PLD at 650 °C, (**c**) BFCO on LAO(100) deposited with MS/PLD at 750 °C. SEM images of films prepared by sputtering, PLD and by hybrid technique: (**d**) MS deposition of BFCO on Si(100), (**e**) PLD deposition of BFCO on Si(100), (**f**) MS/PLD deposition on LAO (100) at 750 °C. The scale bar is 1 μm.

The film grown at 560 °C using the hybrid technique (blue line in Fig. 5) exhibits additional reflections that can be attributed to the presence of Bi-rich phases^36–38^. The film also has a higher RMS roughness value (~12 nm), and AFM shows large particles 196 ± 50 nm in lateral size and 48 ± 19 nm in height uniformly covering the surface (Fig. 7b and Figure S3). For films fabricated using only sputtering, the result is similar: non-epitaxial growth with some secondary phases (BFC*), as indicated in the diffractogram (black line in Fig. 5). AFM data obtained from this film show elongated structures with grains ~290 ± 63 nm in lateral size and 14 ± 5 nm in height (Fig. 7a and Figure S3). The differences between the MS and the PLD processes for BFCO on Si(100) are clearly visible in the SEM images displayed in Fig. 7d and Fig. 7e. The film deposited by PLD contains droplets with a lateral size of ~480 ± 76 nm (Figure S4) and height of 100 nm, whereas the film grown by MS is uniform and much finer-grained. Figure 7f shows the film growth with the hybrid technique on LAO(100). In general the film is uniform, with occasional grains visible on the surface.

**Figure 8:**
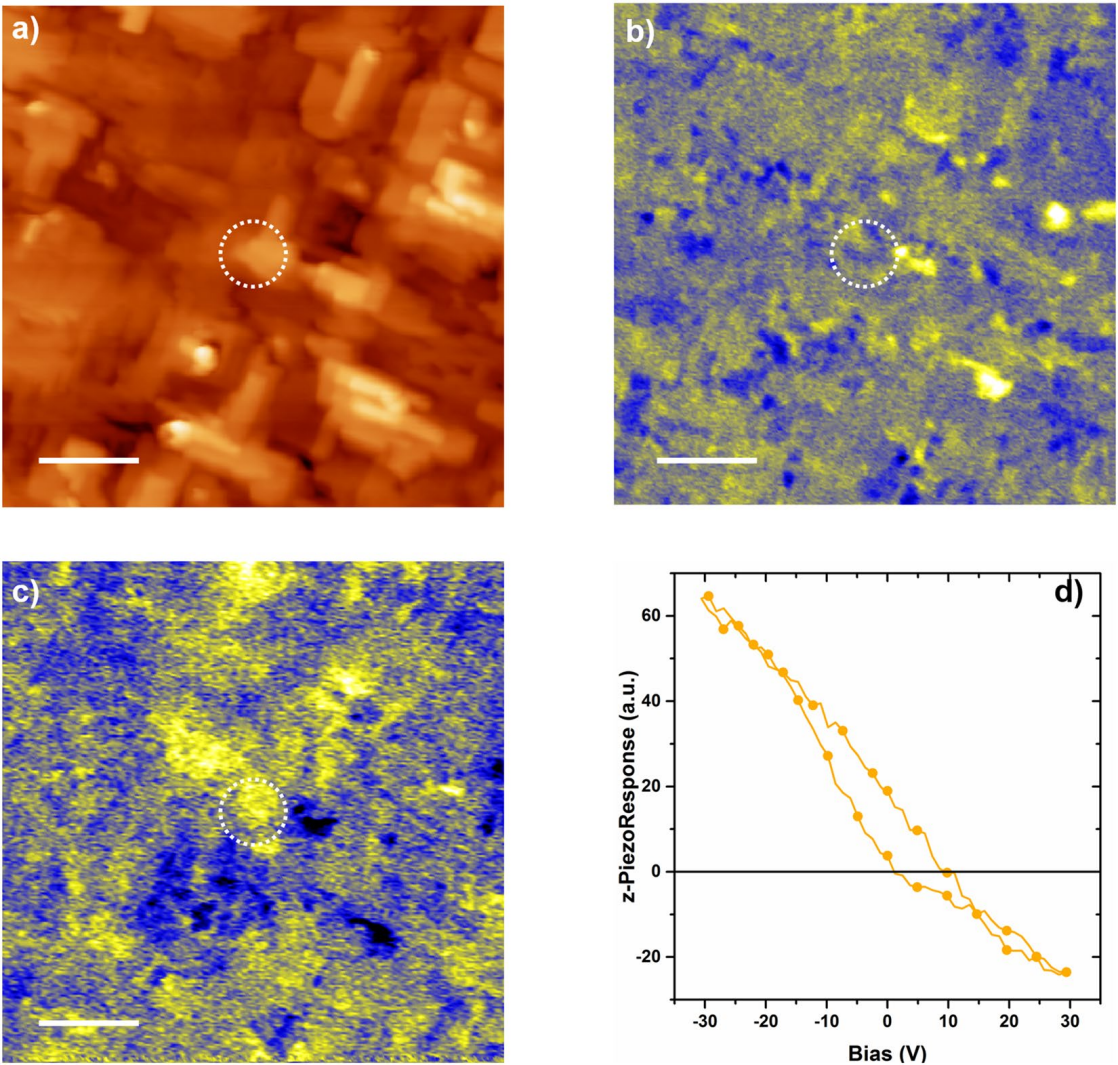
AFM/PFM measurements of BFCO produced with the MS/PLD technique. (**a**) Contact topography AFM image (area 2 μm x 2 μm) (**b**) simultaneously recorded out-of-plane PFM image (**b**) and (**c**) in-plane PFM. The ***z***-range is 25 nm for topography; the z-scale for PFM images is in arbitrary units. The scale bar is 400 nm. (**d**) Piezoresponse hysteresis loop recorded from the area highlighted by the dotted circle.

To verify that the hybrid technique produced a functional BFCO thin film, we investigated its ferroelectric properties by piezoresponse force microscopy (PFM) in several randomly chosen areas. Representative PFM images of the ferroelectric domains in epitaxial BFCO obtained by the hybrid technique are reported in Fig. 8. The out-of-plane and in-plane components of the PFM signal are simultaneously recorded together with topography (Fig. 8a–c). In general, for the out-of-plane measurement (Fig. 8b), yellow areas indicate regions with the z-components of spontaneous polarization oriented upwards while blue areas denote a polarization component oriented in the opposite direction, downwards. In the in-plane measurement, the different colors indicate opposite lateral components of the spontaneous polarization. Figure 8d reports the piezo-response hysteresis loop of the out-of-plane component recorded from the area highlighted in Fig. 8a and b. In Figure S5, we also show the in-plane hysteresis loop recorded on the same area. The existence of hysteresis loops confirm the presence of a switchable ferroelectric polarization, hence confirming the ferroelectric character of the BFCO film produced by the hybrid technique^36, 38^. We carried out a similar investigation on films grown by PLD and MS alone but no piezoresponse was observed (Figure S6), confirming that the hybrid technique was the only deposition approach capable of synthesizing a functional BFCO film.

## Conclusions

We have demonstrated the efficacy of simultaneously combining PLD and MS in a hybrid deposition system for the synthesis of functional materials. TiO_2_ films grown on Si(100) using the hybrid approach exhibit favorable physical properties. When deposited under optimal conditions, they are smoother and denser (~20% more dense for deposition at 5 mTorr) than films produced by either PLD or MS alone. The hybrid technique was also capable of growing epitaxial films of BFCO on LAO(100), suggesting that it could be useful for the controlled synthesis of multifunctional oxides. For both types of materials, the deposition rate of the hybrid technique is 50% higher than either PLD or MS in isolation. Since only minimal changes are required to modify an existing PLD or MS system for hybrid deposition, this approach may be appealing for industrial applications. These initial results indicate that the hybrid PLD/MS approach could be a useful, easily implemented addition to the existing repertoire of film deposition techniques.

### Experimental Details

All experiments were carried out on a Glaze series PLD system commercialized by Plasmionique Inc. with a 1″ Magnion series magnetron. Three types of samples were fabricated: films were grown with (i) PLD alone (KrF Lumonics PM-800 excimer laser, λ = 248 nm, pulse duration = 15.4 ns), (ii) MS alone, (iii) the hybrid system (PLD/MS). TiO_2_ films were deposited on Si(100) at room temperature under three different partial argon pressures: 1, 5 and 40 mTorr. Two different values of laser power were used: 0.56 W and 4.6 W (measured at 20 Hz), and the sputtering power was in the range 35 ÷ 60 W. The repetition rate was fixed at 10 Hz and the deposition duration at 1 hr. Following previous work^35, 36^, BFCO films were deposited on Si(100) and LAO(100) at different argon/oxygen pressures (ratio 3:1, 10 and 70 mTorr) and temperatures (560–750 °C). The laser power (λ = 248 nm) was varied between 0.56 W and 4.6 W (measured at 20 Hz) and the sputtering power was in the range 26 ÷ 60 W. The minimum working pressure for maintaining a stable plasma was found to be 1 mTorr while the minimum laser power was 0.56 W. Under these conditions the minimum power applied on the magnetron for successful generation of a plasma was 35 W.

XRD and XRR were carried out to analyze the crystal quality, film orientation and film thickness using a high-resolution Panalytical X’pert Pro diffractometer equipped with a Cu K_α_ source. The XRR data were fitted using the MOTOFIT add-on^39^ for IGOR Pro data analysis software (WaveMetrics, Inc.), which uses the Abeles matrix method to calculate reflectance in stratified media, allowing for the extraction of parameters such as the roughness of the interfaces and the thickness and the scattering length density of each of the layers in the sample. The reported uncertainty values for the XRR data were generated by MOTOFIT. For a detailed explanation of the method, refer to Supplementary Info.

The average growth rates were calculated from final film thicknesses determined by either XRR, AFM or with a profilometer. At least three measurements were collected using each technique, and the reported uncertainties reflect the spread of values obtained from these measurements.

The morphology of the deposited thin films was characterized using SEM (FE-SEM LEO 1525 microscope) and AFM (Veeco Enviroscope). The RMS roughness values were calculated as an average of different areas (at least three) of 5 × 5 μm^2^. For PFM, an oscillating testing voltage of 0.5 V was applied between the tip and the substrate. Hysteresis measurements were obtained using an auxiliary digital-to-analog converter of the computer-controlled lock-in amplifier by sweeping an additional direct current (DC) bias voltage between −30 V and +30 V.

## Electronic supplementary material

Supplementary Info
