# Supplementary material for: Combined magnetron sputtering and pulsed laser deposition of TiO2 and BFCO thin films
Source: Sci Rep. 2017 May 31;7:2503. doi: 10.1038/s41598-017-02284-0 (PMC5451404; doi:10.1038/s41598-017-02284-0)
Supplement: Supplementary file 1 — Supplementary Info [file 41598_2017_2284_MOESM1_ESM.pdf]

Combined magnetron sputtering and pulsed laser deposition of TiO_2 and BFCO thin films

D. Benetti[1], R. Nouar[2], R. Nechache[4], H. Pepin[1], A. Sarkissian[2], F.Rosei*[1], J. MacLeod*[1,3]

1. INRS Centre for Energy, Materials and Telecommunications, 1650 Boul. Lionel Boulet, J3X 1S2 Varennes, QC, Canada.
2. Plasmionique Inc., 1650 Boul. Lionel Boulet, J3X 1S2 Varennes, QC, Canada.
3. School of Chemistry, Physics, and Mechanical Engineering, Queensland University of Technology, Brisbane 4001, QLD, Australia
4. Département de Génie Electrique, Ecole de technologie supérieure, 1100 rue Notre-Dame Ouest, Montréal, QC H3C 1K3, Canada

*Corresponding Authors: rosei@emt.inrs.ca, jennifer.macleod@qut.edu.au

SUPPORTING INFORMATION

MOTOFIT SIMULATION OF XRR DATA

Motofit is an add-on package to IGOR Pro data analysis software (WaveMetrics, Inc.), created for interpreting reflectivity data. Using known information about the sample, MOTOFIT fits reflectivity data according to a model based on Abeles Formalism. By varying parameters of the theoretical model, a close fit to the XRR data can be achieved. The freely varying parameters, including roughness of the interfaces and the thickness and the scattering length density of each of the layers in the sample, can then be extracted.

For our data, we used a three layer model that included an intermediate layer of silicon oxide between the Si wafer and the TiO_2 film (Figure S1).

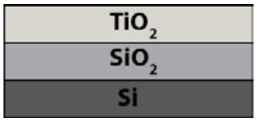

Figure S1: Three layer model used for fitting the XRR data.

The SLD values for Si and SiO_2 were fixed to their nominal values, $20.1 \times 10^{-6} Å^{-2}$ and $18.76 \times 10^{-6} Å^{-2}$ respectively (values obtained from www.ncnr.nist.gov/resources/activation/). The SLD value for the TiO_2 was free to vary around the nominal value of $34.46 \times 10^{-6} Å^{-2}$ All the other free parameters were allowed to vary without constraints.

The fitting used was a Genetic+LM algorithm, which it uses the Levenburg-Marquardt method for minimizing the χ^2 value. The Genetic Optimization algorithm is capable of finding global minima in the χ^2 function. After this first fit, the parameters were optimized using a Levenberg-Marquardt method. The convergence was reached by setting a fit tolerance value of 0.01: if the fractional decrease in the best χ^2 value was less than this value then the fit was terminated. (*Journal of Applied Crystallography, 39, 273-276, 2006*)

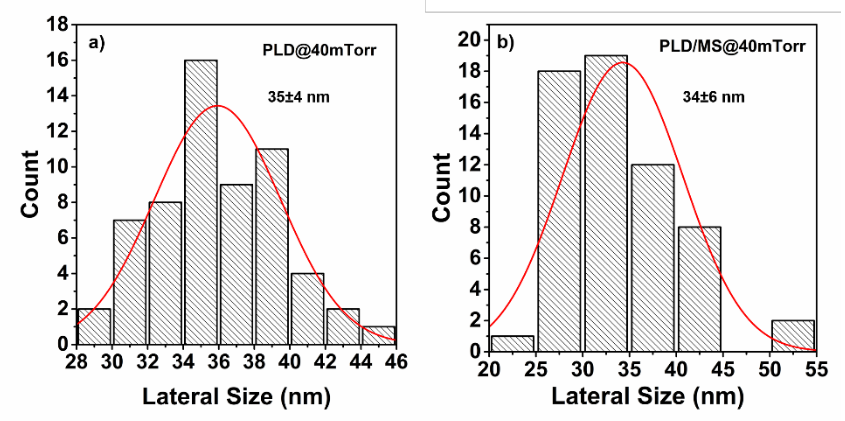

Figure S2: Grain size distribution of the TiO_2 films at 40 mTorr a) deposited with PLD technique b) deposited with the hybrid technique

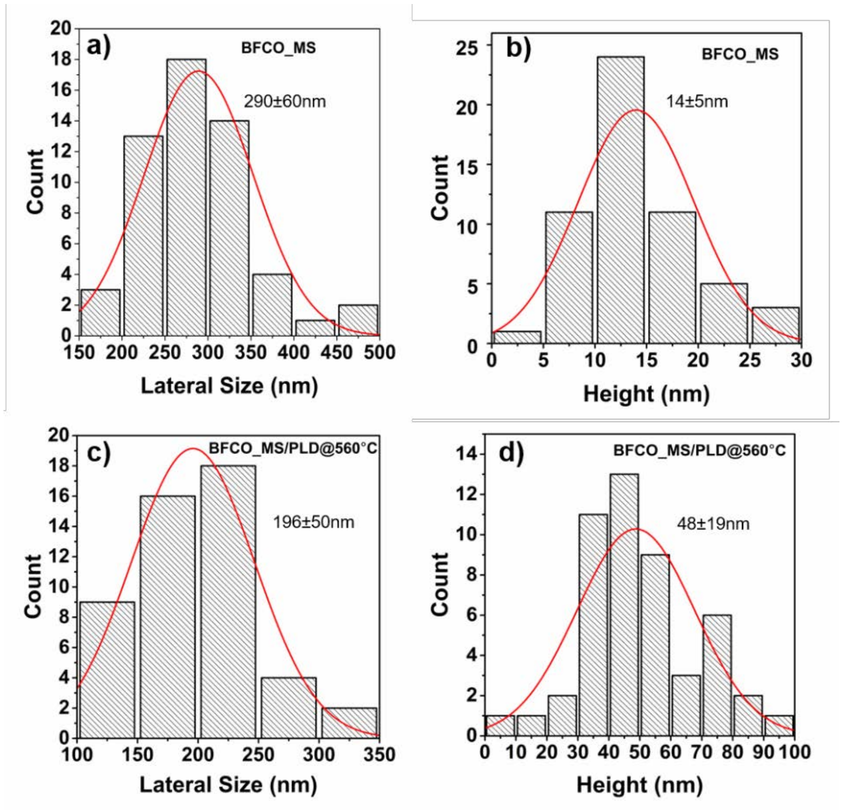

Figure S3: Grain size distribution of the BFCO films a and b) deposited with MS technique c and d) deposited with the hybrid technique at 560 °C on LAO(100).

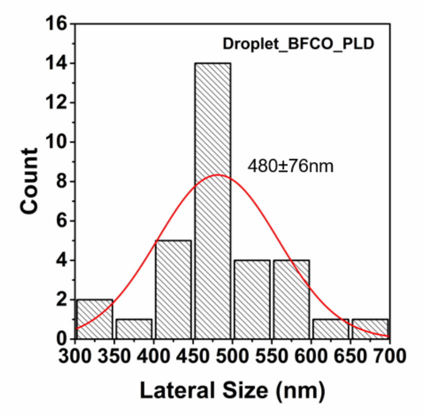

Figure S4: Droplet size distribution of the BFCO film deposited with PLD technique on Si(100).

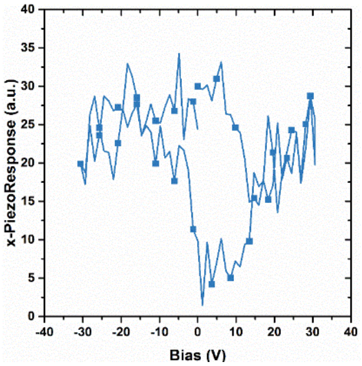

Figure S5: In plane piezoresponse of the BFCO film deposited with the hybrid technique.

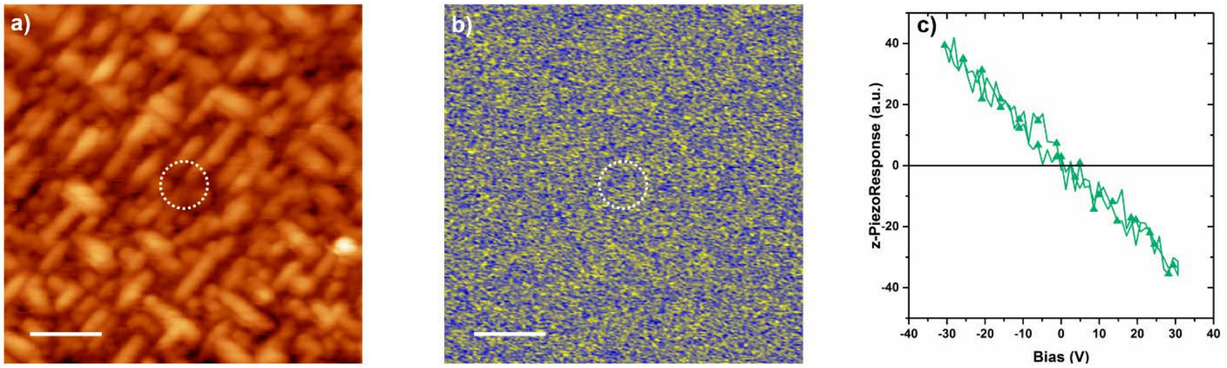

Figure S6: AFM/PFM measurements of BFCO produced with the MS technique. a) Contact topography AFM image (area 2um x 2 um) b) simultaneously recorded out-of-plane PFM image. The *z*-range is 25 nm for topography; the z-scale for PFM image is in arbitrary units. Scale bar is 400nm. c) Piezo-response hysteresis loop recorded from the area highlighted by the dotted circle.

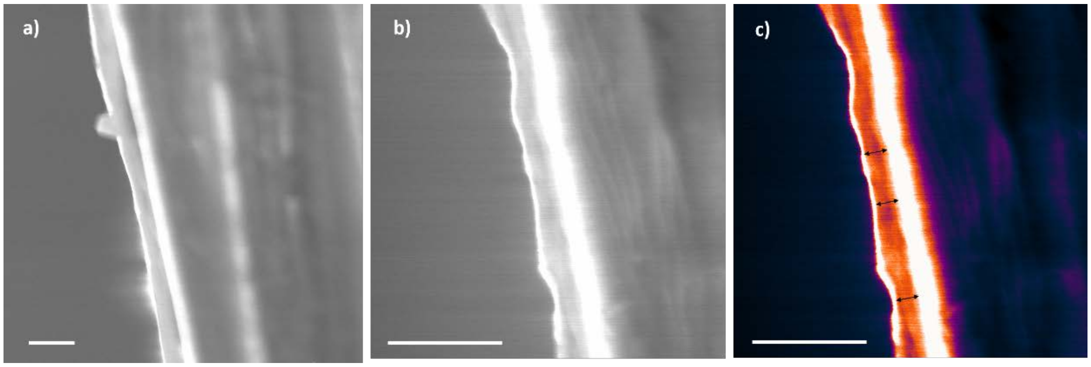

Figure S7:Cross section of TiO_2 film on Si(100) obtained with the hybrid technique at different zooms (a) 10000x and (b) 25000x. (c) highlight of the TiO_2 thickness. The average value is 144nm, value closed to the one measured with XRR (131nm).
